# Supplementary material for: Genes encoding cytochrome P450 monooxygenases and glutathione S-transferases associated with herbicide resistance evolved before the origin of land plants
Source: PLoS One. 2023 Feb 17;18(2):e0273594. doi: 10.1371/journal.pone.0273594 (PMC9937507; doi:10.1371/journal.pone.0273594)
Supplement: S1 Fig — Diagram of a typical CYP protein showing recognisable amino acid sites. GST G-site and H-site locations in this figure are based on the crystal structure of TaGSTU4. (PDF) [file pone.0273594.s001.pdf]

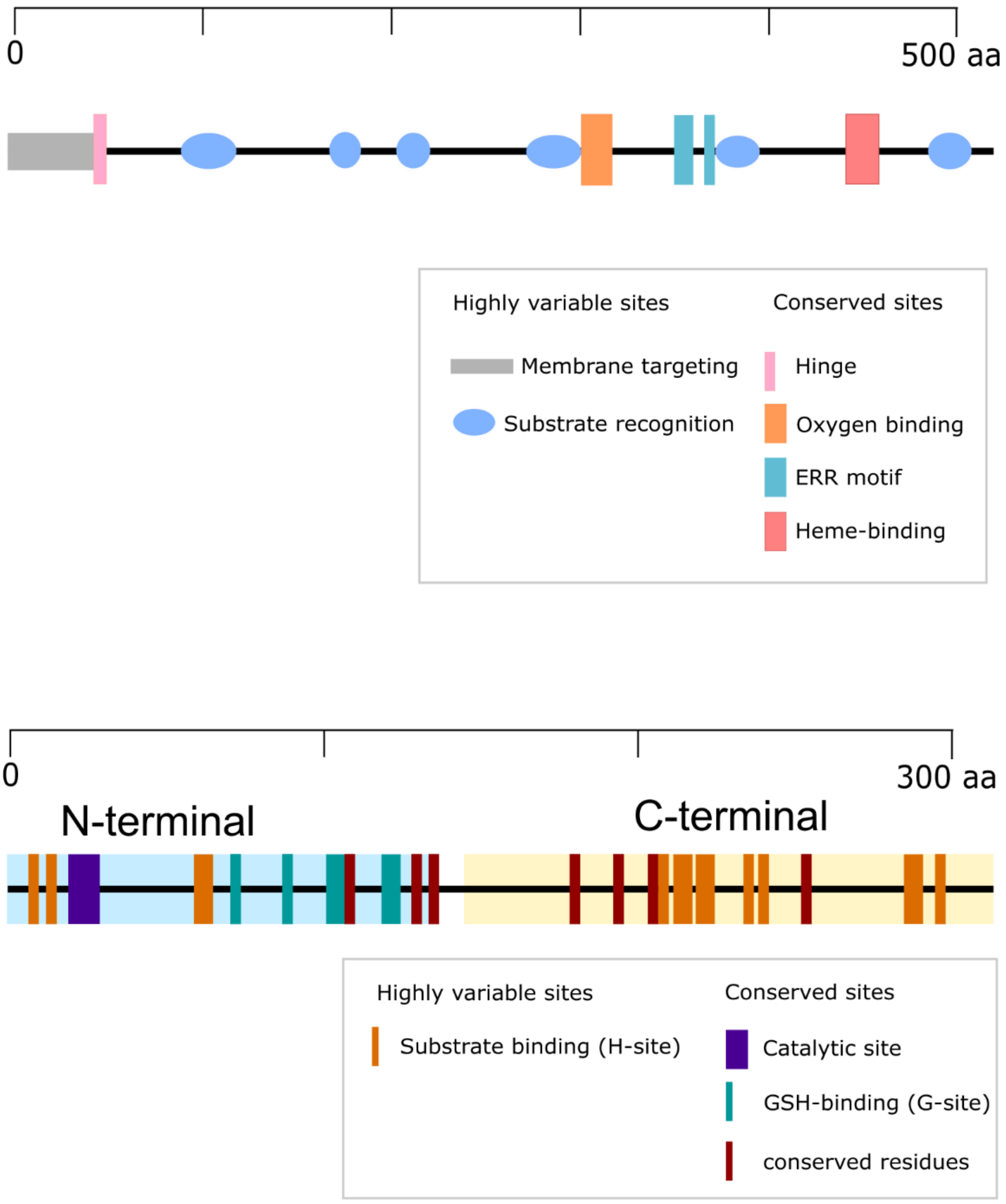

Fig S1. Protein features of cytochrome P450s and glutathione *S*-transferases in plants. Diagram of a typical CYP and GST protein showing recognisable amino acid sites. GST G-site and H-site locations in this figure are based on the crystal structure of TaGSTU4-4 [24].
