# Supplementary material for: Genes encoding cytochrome P450 monooxygenases and glutathione S-transferases associated with herbicide resistance evolved before the origin of land plants
Source: PLoS One. 2023 Feb 17;18(2):e0273594. doi: 10.1371/journal.pone.0273594 (PMC9937507; doi:10.1371/journal.pone.0273594)
Supplement: S2 Fig — Unrooted cladograms of maximum likelihood (ML) analysis conducted by PHyML 3.0 [75] using an estimated gamma distribution parameter, the LG+G+F model of amino acid substitution and a Chi2-based approximate likelihood ratio (aLRT) test. CYP (A) and GST (B) sequences were aligned in MAFFT and trimmed with the automatic trimming software trimAl using the automatic modes -strictplus, -strict, -gappyout or by manual trimming. Branches are coloured to show the different CYP clans or GST classes. aLRT Support values for some of the clades are shown for comparison. (PDF) [file pone.0273594.s002.pdf]

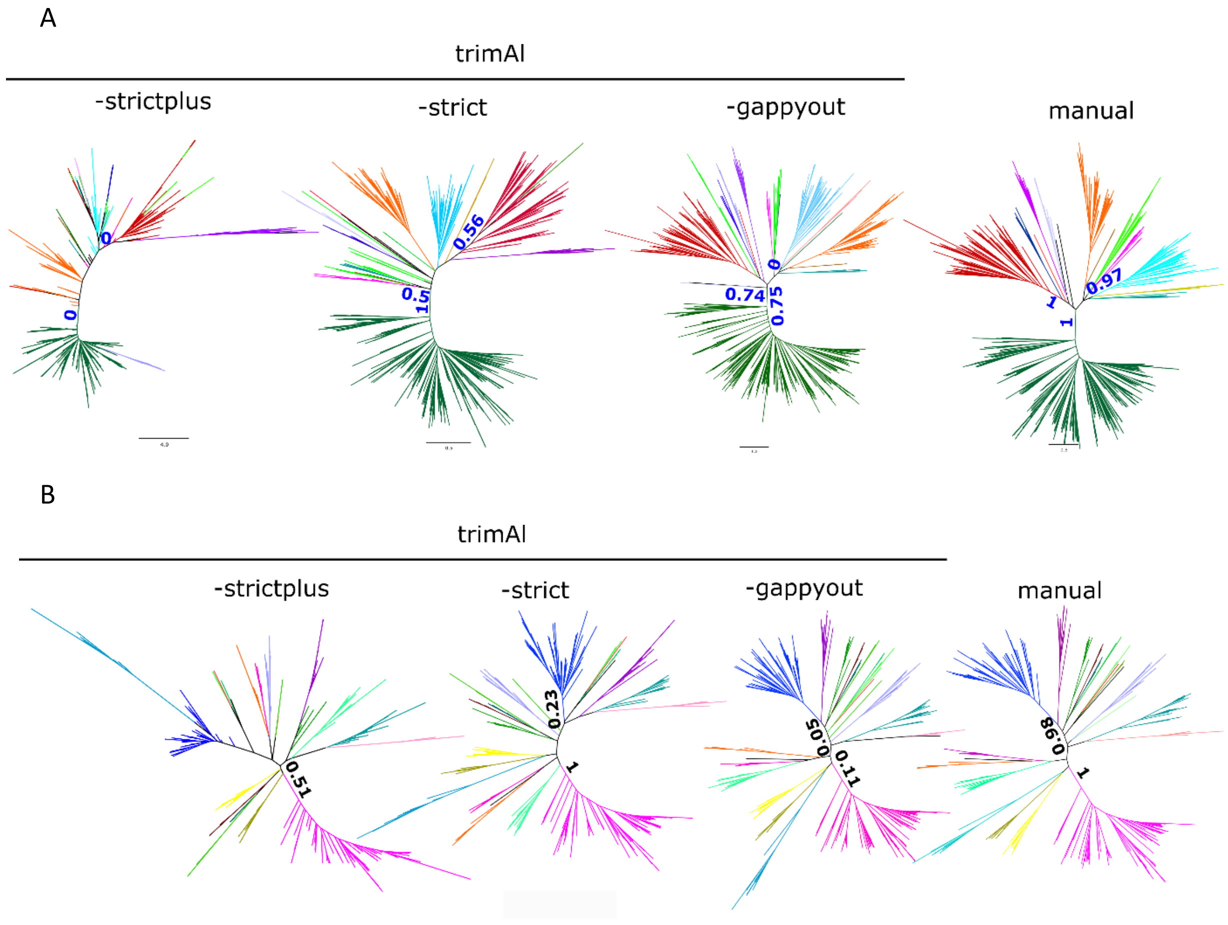

Fig S2. Plant CYP and GST phylogenetic analysis using automatic and manual trimming approaches.

Unrooted cladograms of maximum likelihood (ML) analysis conducted by PHyML 3.0 [58] using an estimated gamma distribution parameter, the LG+G+F model of amino acid substitution and a Chi^2-based approximate likelihood ratio (aLRT) test. CYP (A) and GST (B) sequences were aligned in MAFFT and trimmed with the automatic trimming software trimAl using the automatic modes -strictplus, -strict, -gappyout or by manual trimming. Branches are coloured to show the different CYP clans or GST classes. aLRT Support values for some of the clades are shown for comparison.
