# Supplementary material for: Genes encoding cytochrome P450 monooxygenases and glutathione S-transferases associated with herbicide resistance evolved before the origin of land plants
Source: PLoS One. 2023 Feb 17;18(2):e0273594. doi: 10.1371/journal.pone.0273594 (PMC9937507; doi:10.1371/journal.pone.0273594)
Supplement: S3 Fig — Representative sequences from each plant species in this study are included for each clan. Sequences were aligned in MAFFT using the FFT-NS-i algorithm. The locations of the substrate recognition sites are based on those identified in Arabidopsis CYPs in [37]. The absolutely conserved cysteine that binds the heme within the heme-binding domain is marked with an asterisk. (PDF) [file pone.0273594.s003.pdf]

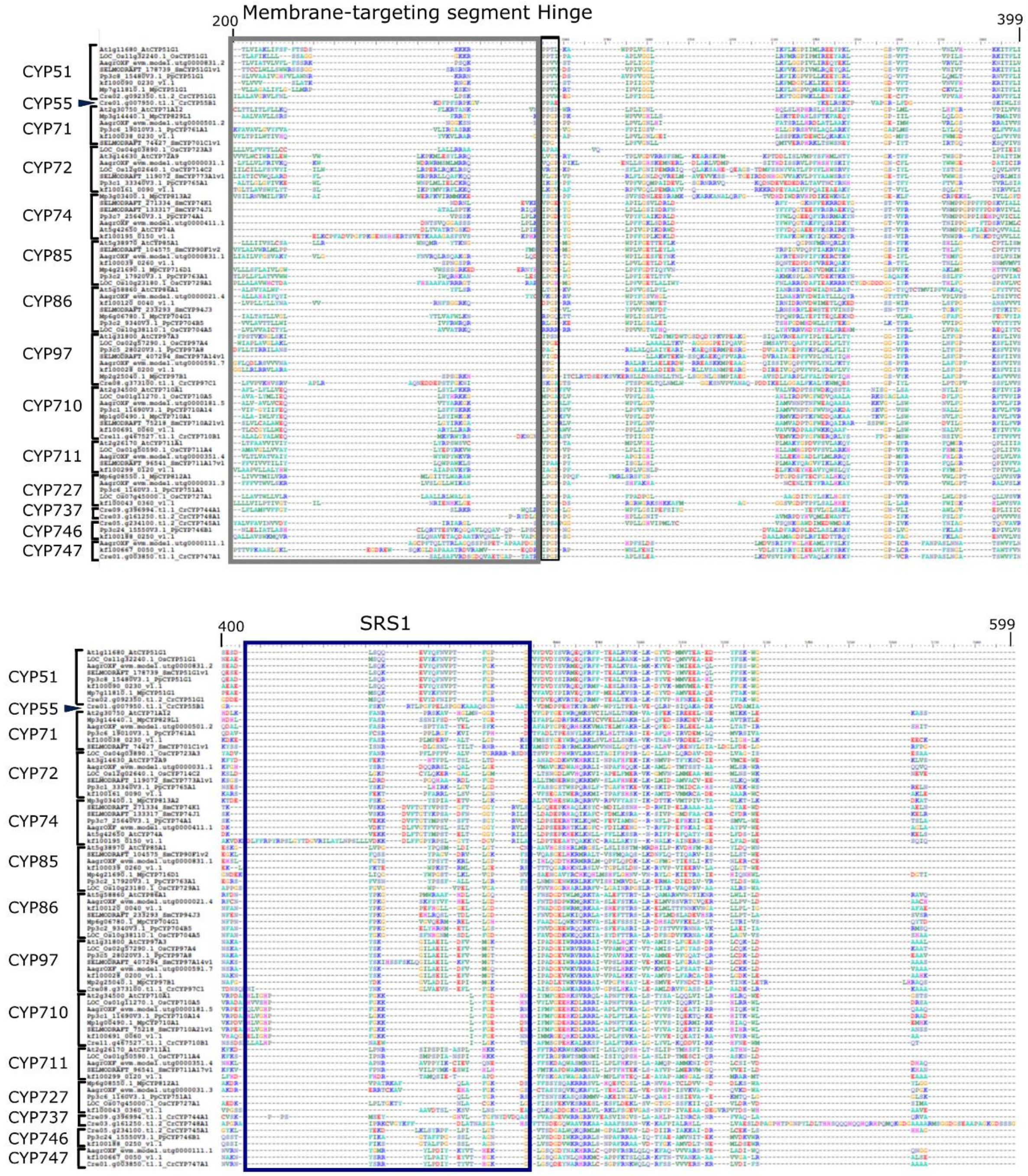
200
Membrane-targeting segment
Hinge
399
CYP51
CYP55
CYP71
CYP72
CYP74
CYP85
CYP86
CYP97
CYP710
CYP711
CYP727
CYP737
CYP746
CYP747
400
SRS1
599
CYP51
CYP55
CYP71
CYP72
CYP74
CYP85
CYP86
CYP97
CYP710
CYP711
CYP727
CYP737
CYP746
CYP747

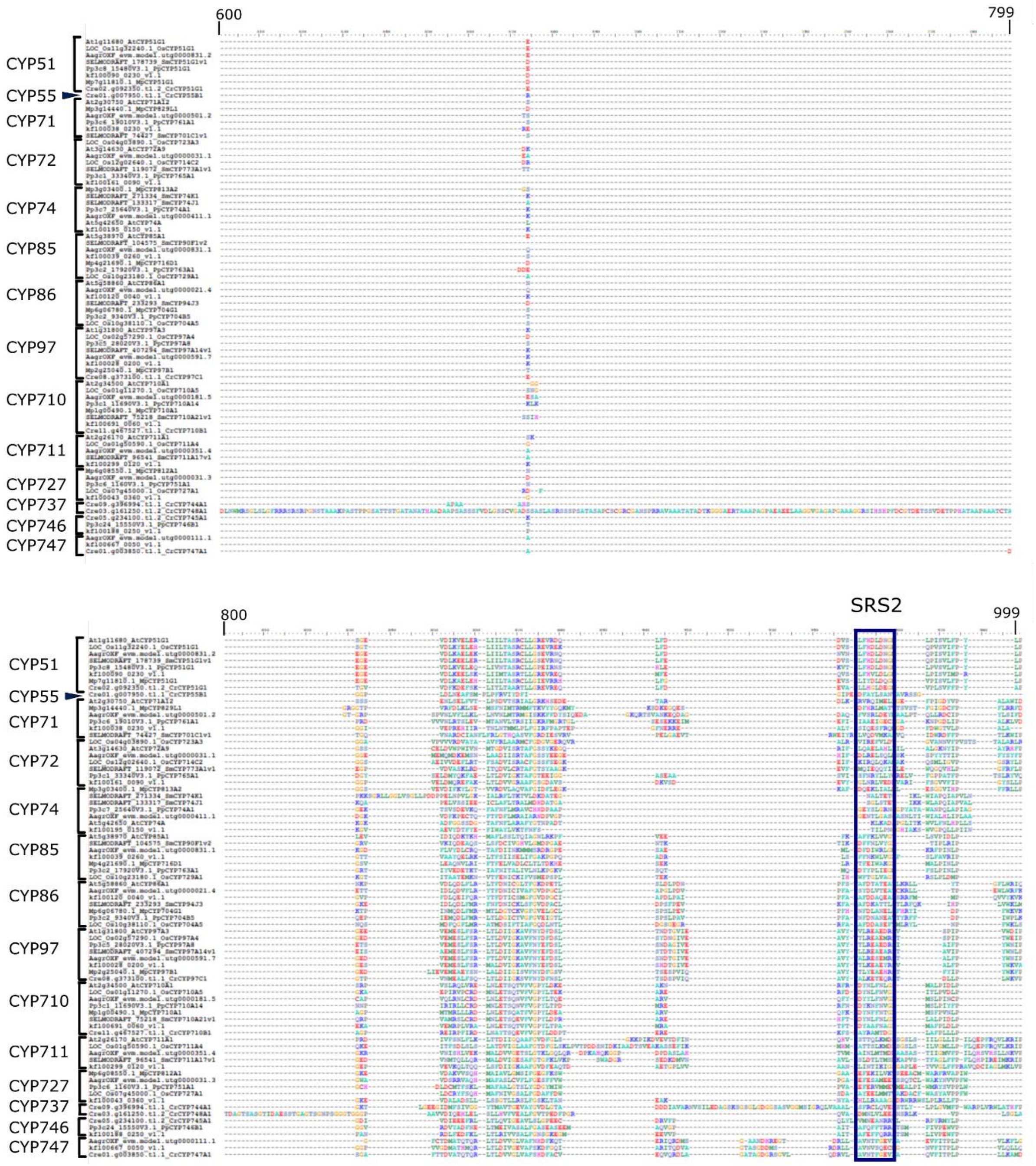
600
799
CYP51
CYP55
CYP71
CYP72
CYP74
CYP85
CYP86
CYP97
CYP710
CYP711
CYP727
CYP737
CYP746
CYP747
800
999
SRS2

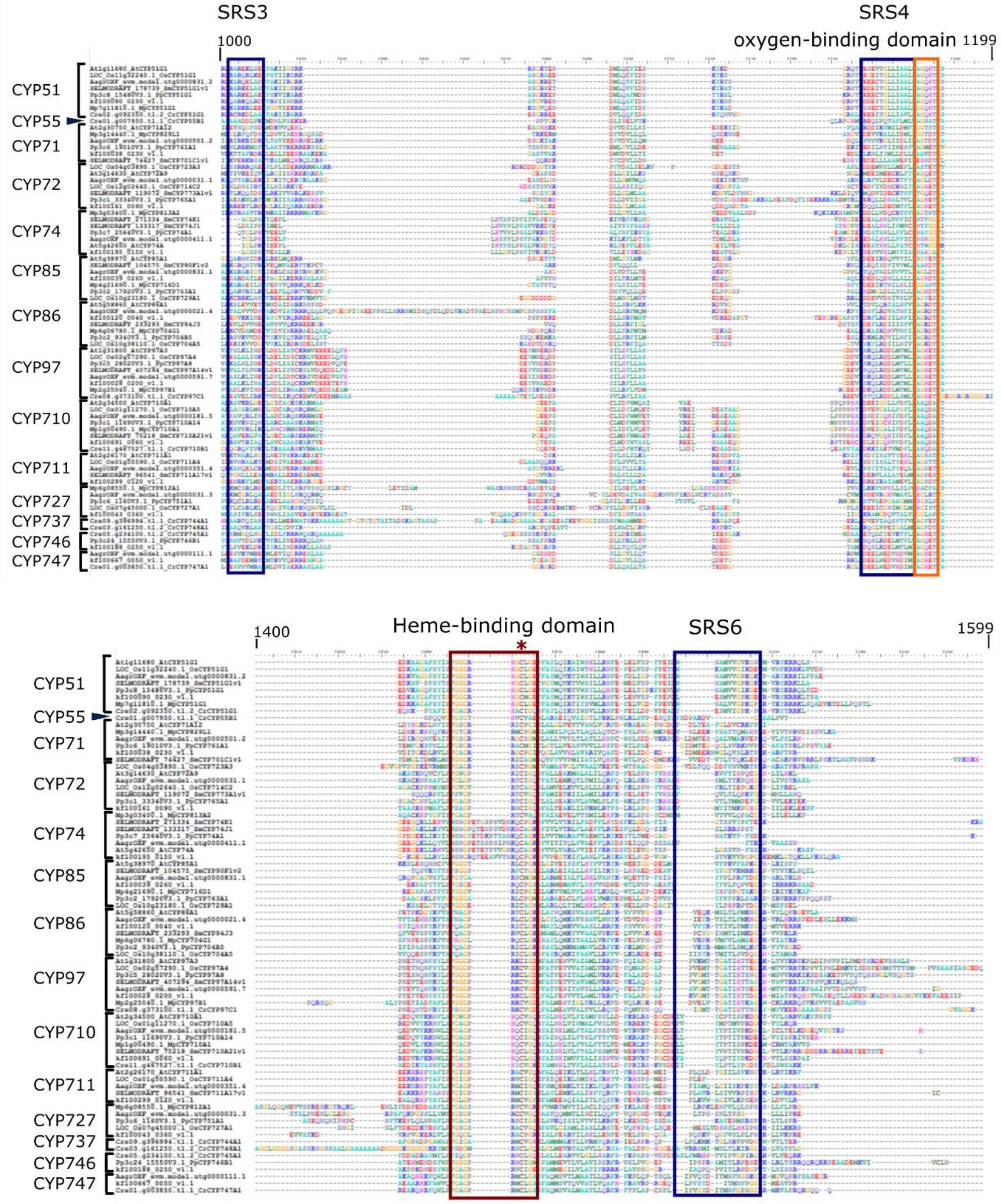
SRS3
SRS4
1000
oxygen-binding domain
1199
CYP51
CYP55
CYP71
CYP72
CYP74
CYP85
CYP86
CYP97
CYP710
CYP711
CYP727
CYP737
CYP746
CYP747
1400
Heme-binding domain
SRS6
1599
*
CYP51
CYP55
CYP71
CYP72
CYP74
CYP85
CYP86
CYP97
CYP710
CYP711
CYP727
CYP737
CYP746
CYP747

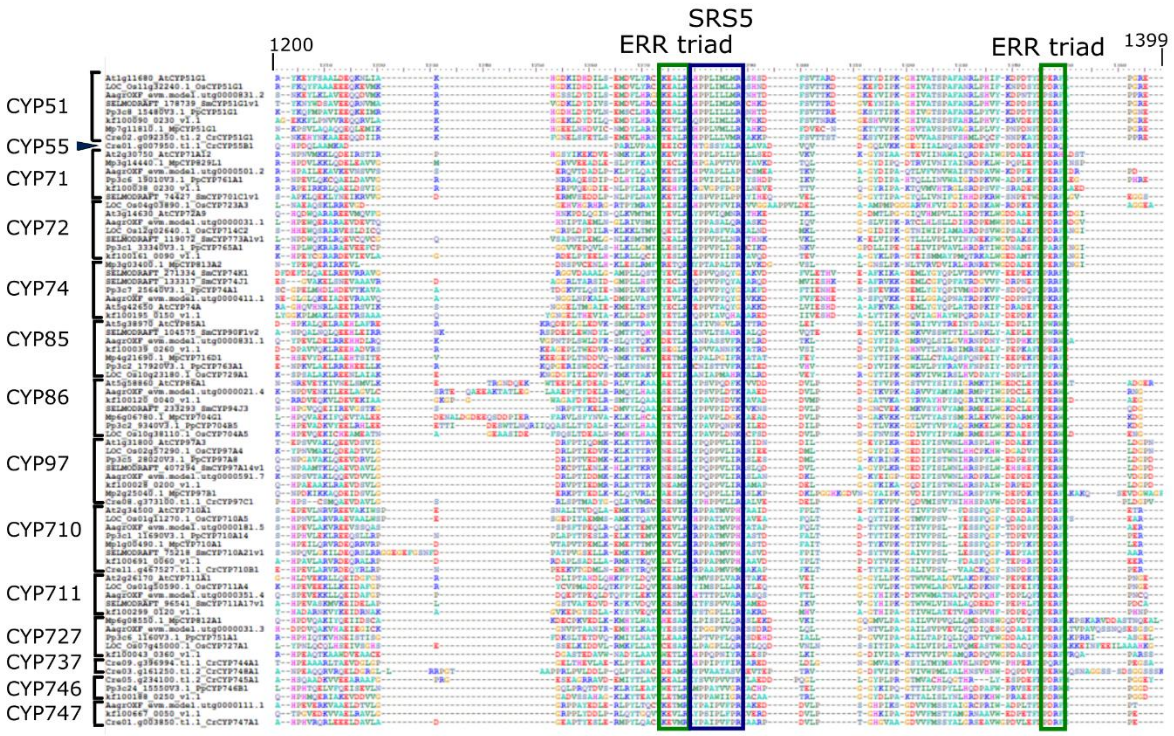

Fig S3. Untrimmed amino acid alignment of representative CYP proteins from each clan showing the location of conserved CYP domains. Representative sequences from each plant species in this study are included for each clan. Sequences were aligned in MAFFT using the FFT-NS-i algorithm. The locations of the substrate recognition sites are based on those identified in Arabidopsis CYPs in [37]. The absolutely conserved cysteine that binds the heme within the heme-binding domain is marked with an asterisk.
