# Supplementary figures and images for: Genes encoding cytochrome P450 monooxygenases and glutathione S-transferases associated with herbicide resistance evolved before the origin of land plants
Source: PLoS One. 2023 Feb 17;18(2):e0273594. doi: 10.1371/journal.pone.0273594 (PMC9937507; doi:10.1371/journal.pone.0273594)

## N-terminal domain

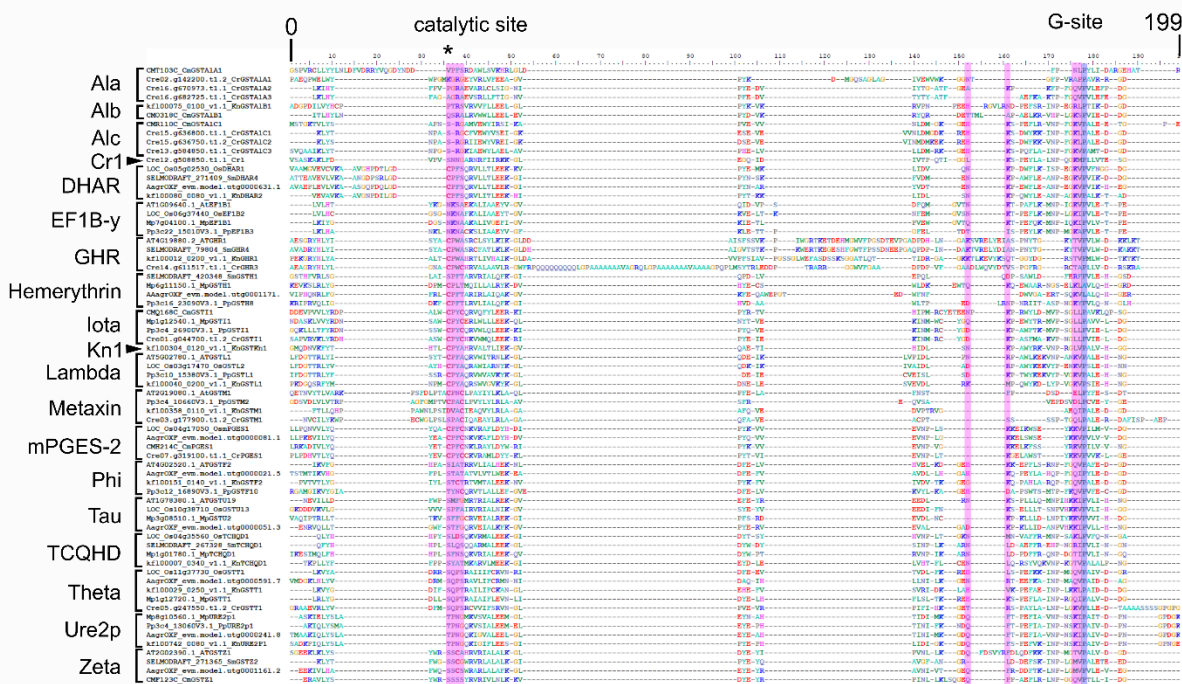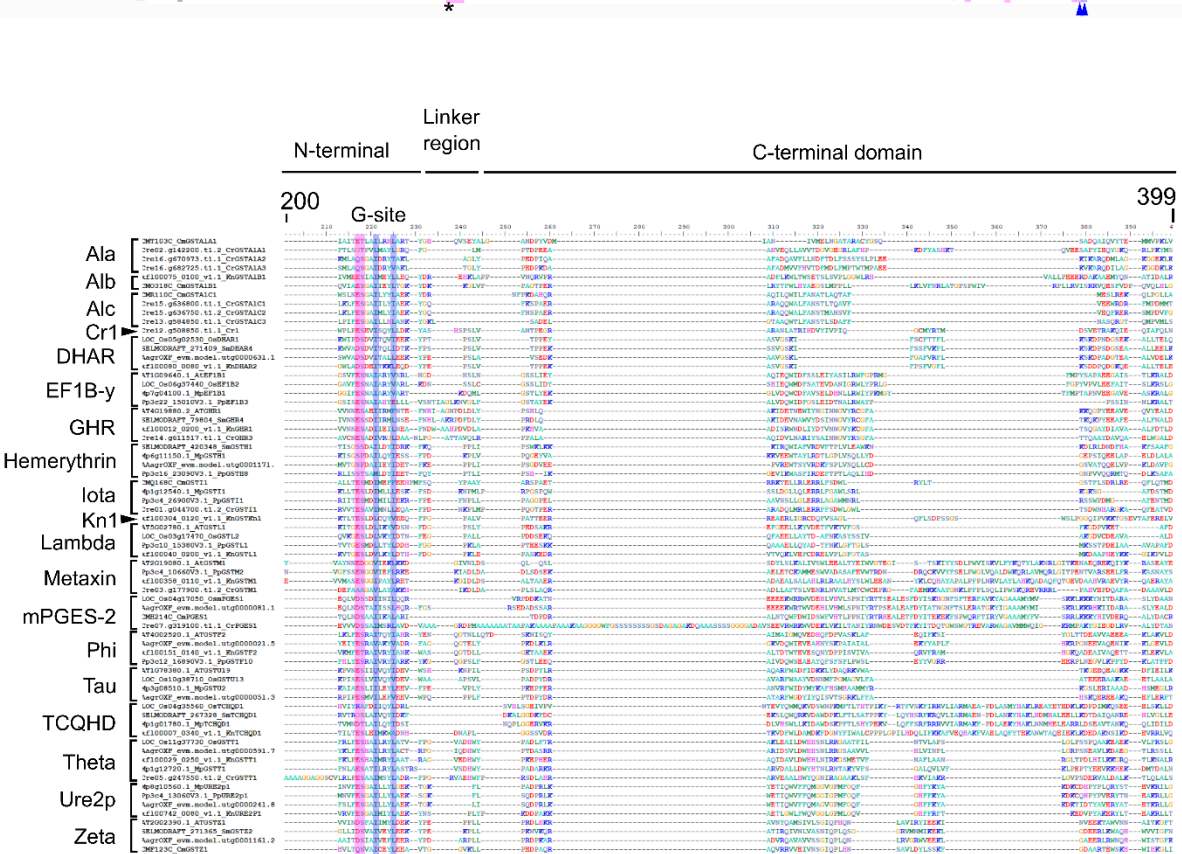

Supplement: S4 Fig — Sequences were aligned in MAFFT using the FFT-NS-i algorithm. Four representative sequences from different species are shown for each GST class. The location of the putative catalytic residue is indicated with an asterisk. Sites that bind GSH (G-sites) are indicated in solid pink. Residues conserved in at least 80% of samples are indicated by blue arrows. GSTHs and GSTIs have large domains that extend past the C-terminal domain end which haven’t been included in the figure. G-site residues are based on the crystal structure of TaGSTU4 [24]. (PDF) [file pone.0273594.s004.pdf]
