# Supplementary material for: Genes encoding cytochrome P450 monooxygenases and glutathione S-transferases associated with herbicide resistance evolved before the origin of land plants
Source: PLoS One. 2023 Feb 17;18(2):e0273594. doi: 10.1371/journal.pone.0273594 (PMC9937507; doi:10.1371/journal.pone.0273594)
Supplement: S1 Table — Numbers of CYP proteins in each clan, excluding pseudogenes. At, Arabidopsis thaliana; Os, Oryza sativa; Sm, Selaginella moellendorffii; Aa, Anthoceros agrestis; Pp, Physcomitrium patens; Mp, Marchantia polymorpha; Kn, Klebsormidium nitens; Cr, Chlamydomonas reinhardtii; Cm, Cyanidioschyzon merolae. (PDF) [file pone.0273594.s005.pdf]

Table S1. Cytochrome P450 clans and gene numbers in green plants and red algae.

CYP Clan	*At*	*Os*	*Sm*	*Aa*	*Pp*	*Mp*	*Kn*	*Cr*	*Cm*
51	1	8	1	1	1	1	3	1	1
55	0	0	0	0	0	0	0	1	0
71	148	163	98	59	38	68	3	0	0
72	18	32	37	13	6	8	5	0	0
74	2	5	12	1	3	2	1	0	0
85	28	30	32	53	4	19	2	0	0
86	33	41	14	9	10	11	3	0	0
97	3	3	3	3	3	3	3	4	0
710	4	4	1	2	2	2	1	1	2
711	1	4	1	1	0	0	4	13	0
727	0	1	0	1	1	1	2	0	0
737	0	0	0	0	0	0	0	16	0
741	0	0	0	0	0	0	0	2	0
746	0	0	0	0	1	0	1	1	0
747	0	0	0	1	0	0	1	1	0
Cm1	0	0	0	0	0	0	0	0	1
Cm2	0	0	0	0	0	0	0	0	1
Total	238	291	199	144	69	115	29	40	5

Numbers of CYP proteins in each clan, excluding pseudogenes. *At, Arabidopsis thaliana; Os, Oryza sativa; Sm, Selaginella moellendorffii; Aa, Anthoceros agrestis; Pp, Physcomitrium patens; Mp, Marchantia polymorpha; Kn, Klebsormidium nitens; Cr, Chlamydomonas reinhardtii; Cm, Cyanidioschyzon merolae.*
