# Supplementary material for: Genes encoding cytochrome P450 monooxygenases and glutathione S-transferases associated with herbicide resistance evolved before the origin of land plants
Source: PLoS One. 2023 Feb 17;18(2):e0273594. doi: 10.1371/journal.pone.0273594 (PMC9937507; doi:10.1371/journal.pone.0273594)
Supplement: S2 Table — Numbers of GST proteins in each clan, excluding pseudogenes. At, Arabidopsis thaliana; Os, Oryza sativa; Sm, Selaginella moellendorffii; Aa, Anthoceros agrestis; Pp, Physcomitrium patens; Mp, Marchantia polymorpha; Kn, Klebsormidium nitens; Cr, Chlamydomonas reinhardtii; Cm, Cyanidioschyzon merolae. (PDF) [file pone.0273594.s006.pdf]

Table S2. Glutathione-S-transferase classes and gene numbers in green plants and red algae.

GST Class	***At***	***Os***	***Sm***	***Aa***	***Pp***	***Mp***	***Kn***	***Cr***	***Cm***
AlA	0	0	0	0	0	0	0	7	1
AlB	0	0	0	0	0	0	1	0	1
AlC	0	0	0	0	0	0	0	3	1
Cr1	0	0	0	0	0	0	0	1	0
DHAR	3	2	4	2	3	2	2	1	0
EF1B-γ	2	2	1	1	4	1	1	0	1
GHR	4	2	5	2	3	2	3	3	1
Hemerythrin	0	0	1	1	8	1	0	0	0
Iota	0	0	1	1	1	1	1	1	1
Kn1	0	0	0	0	0	0	1	0	0
Lambda	3	3	0	0	1	0	1	0	0
Metaxin	1	1	1	1	2	1	1	1	0
mPGES2	1	1	1	1	0	1	1	1	2
Phi	13	19	1	11	10	18	3	0	0
Tau	28	49	34	1	0	2	3	0	0
TCHQD	1	1	1	0	5	2	3	0	0
Theta	3	1	3	1	3	2	1	1	0
Ure2p	0	0	2	2	1	1	1	0	0
Zeta	2	4	2	2	1	1	1	0	1
Total	61	85	57	26	42	35	24	19	9

Numbers of GST proteins in each clan, excluding pseudogenes. *At, Arabidopsis thaliana; Os, Oryza sativa; Sm, Selaginella moellendorffii; Aa, Anthoceros agrestis; Pp, Physcomitrium patens; Mp, Marchantia polymorpha; Kn, Klebsormidium nitens; Cr, Chlamydomonas reinhardtii; Cm, Cyanidioschyzon merolae.*
