# Supplementary material for: Genes encoding cytochrome P450 monooxygenases and glutathione S-transferases associated with herbicide resistance evolved before the origin of land plants
Source: PLoS One. 2023 Feb 17;18(2):e0273594. doi: 10.1371/journal.pone.0273594 (PMC9937507; doi:10.1371/journal.pone.0273594)
Supplement: S3 Table — (PDF) [file pone.0273594.s007.pdf]

S3 Table. Candidate NTSR CYPs belong to several CYP clans.

Clan	Sub-family	Gene name	Evidence	Herbicide chemical class	genes per clan	References
71	71A	AmCYP71A (blackgrass)	up-regulated in NTSR plants compared to sensitive plants	DIM, Dinitroaniline, FOP, Sulfonylurea	28	[1]
	71A	CbCYP71A26 (hairy fleabane)	up-regulated in NTSR plants compared to sensitive plants	Glycine		[2]
	71B	AmCYP71B3 (blackgrass)	up-regulated in NTSR plants compared to sensitive plants	DIM, Dinitroaniline, FOP, Sulfonylurea		[1]
	71B	CbCYP71B7 (hairy fleabane)	up-regulated in NTSR plants compared to sensitive plants	Glycine		[2]
	71B	CbCYP72B7 (hairy fleabane)	up-regulated in NTSR plants compared to sensitive plants	Glycine		[2]
	71C	ZmCYP71C2 (maize)	up-regulated in NTSR plants compared to sensitive plants	Sulfonylurea		[3]
	71C	ZmCYP71C3v2 (maize)	up-regulated in NTSR plants compared to sensitive plants	Sulfonylurea		[3]
	71C	ZmCYP71C4 (maize)	up-regulated in NTSR plants compared to sensitive plants	Sulfonylurea		[3]
	71D	BsCYP71D7 (American slough grass)	up-regulated in NTSR plants compared to sensitive plants	FOP		[4]
	71D	BsCYP71D10 (American slough grass)	up-regulated in NTSR plants compared to sensitive plants	FOP		[4]
	71K	EpCYP71K2 (barnyard grass)	up-regulated in NTSR plants compared to sensitive plants	Pyrimidinyl benzoate		[5]
	75B	MaCYP75B2 (giant chickweed)	up-regulated in NTSR plants compared to sensitive plants	Sulfonylurea		[6]
	76C	MaCYP76C1 (giant chickweed)	up-regulated in NTSR plants compared to sensitive plants	Sulfonylurea		[7]
	76C	CbCYP76C2 (hairy fleabane)	up-regulated in NTSR plants compared to sensitive plants	Glycine		[2]
	78A	CbCYP78A-1 (hairy fleabane)	up-regulated in NTSR plants compared to sensitive plants	Glycine		[2]
	78A	CbCYP78A-2 (hairy fleabane)	up-regulated in NTSR plants compared to sensitive plants	Glycine		[2]
	81A	AmCYP81A2 (blackgrass)	up-regulated in NTSR plants compared to sensitive plants	DIM, Dinitroaniline, FOP, Sulfonylurea		[8]
	81A	AmCYP81A3 (blackgrass)	up-regulated in NTSR plants compared to sensitive plants	DIM, Dinitroaniline, FOP, Sulfonylurea		[8]
	81A	AmCYP81A4 (blackgrass)	up-regulated in NTSR plants compared to sensitive plants	DIM, Dinitroaniline, FOP, Sulfonylurea		[8]
	81A	ZmCYP81A9 (maize)	up-regulated in NTSR plants compared to sensitive plants	Sulfonylurea		[3]
	81B	LrCYP81B1 (annual ryegrass)	up-regulated in NTSR plants compared to sensitive plants	Sulfonylurea		[9]
	81D	AmCYP81D (blackgrass)	up-regulated in NTSR plants compared to sensitive plants	DIM, Dinitroaniline, FOP, Sulfonylurea		[1]
	82A	CbCYP82A3 (hairy fleabane)	up-regulated in NTSR plants compared to sensitive plants	Glycine		[2]
	82A	82A3-like (purple morning glory)	up-regulated in NTSR plants compared to sensitive plants	Glycine		[10]
	82A	CbCYP82A4 (hairy fleabane)	up-regulated in NTSR plants compared to sensitive plants	Glycine		[2]
	92A	ZmCYP92A1 (maize)	up-regulated in NTSR plants compared to sensitive plants	Sulfonylurea		[3]
	93A	CbCYP93A3 (hairy fleabane)	up-regulated in NTSR plants compared to sensitive plants	Glycine		[2]
	99A	BsCYP99A2 (American slough grass)	up-regulated in NTSR plants compared to sensitive plants	FOP		[4]
72	72A	LrCYP72A1 (annual ryegrass)	up-regulated in NTSR plants compared to sensitive plants	Sulfonylurea	11	[9]
	72A	LrCYP72A2 (annual ryegrass)	up-regulated in NTSR plants compared to sensitive plants	Sulfonylurea		[9]
	72A	LrCYP72A1 (annual ryegrass)	up-regulated in NTSR plants compared to sensitive plants	FOP		[11]
	72A	LrCYP72A2 (annual ryegrass)	up-regulated in NTSR plants compared to sensitive plants	FOP		[11]
	72A	ZmCYP72A5 (maize)	up-regulated in NTSR plants compared to sensitive plants	Sulfonylurea		[3]
	72A	ApCYP72A219 (palmers amaranth)	up-regulated in NTSR plants compared to sensitive plants	Glycine		[12]
	72A	BhCYP72A14 (hybridous Mediterranean false brome)	up-regulated in NTSR plants compared to sensitive plants	DEN		[13]
	72A	BhCYP72A15 (hybridous Mediterranean false brome)	up-regulated in NTSR plants compared to sensitive plants	DEN		[13]

S3 Table. Candidate NTSR CYPs belong to several CYP clans (continued).

Clan	Sub-family	Gene name	Evidence	Herbicide chemical class	genes per clan	References
72	72A	EpCYP72A254 (barnyard grass)	up-regulated in NTSR plants compared to sensitive plants	Pyrimidinyl benzoate		[5]
	734A	MaCYP734A1 (giant chickweed)	up-regulated in NTSR plants compared to sensitive plants	Sulfonylurea		[7]
	749A	GhCYP749A16 (cotton)	up-regulated in NTSR plants compared to sensitive plants	Sulfonylurea		[14]
74	74A	MaCYP74A (giant chickweed)	up-regulated in NTSR plants compared to sensitive plants	Sulfonylurea	1	[6]
85	87A	BsCYP87A3 (American slough grass)	up-regulated in NTSR plants compared to sensitive plants	FOP	1	[15]
86	86B	MaCYP86B1 (giant chickweed)	up-regulated in NTSR plants compared to sensitive plants	Sulfonylurea	5	[7]
	86B	BcCYP86B1 (American slough grass)	up-regulated in NTSR plants compared to sensitive plants	Sulfonylurea		[16]
	94A	AaCYP94A2 (shortawn foxtail)	up-regulated in NTSR plants compared to sensitive plants	Sulfonylurea		[17]
	94A	CbCYP94A1 (hairy fleabane)	up-regulated in NTSR plants compared to sensitive plants	Glycine		[2]
	96A	DsCYP96A13 (flixweed)	up-regulated in NTSR plants compared to sensitive plants	Sulfonylurea		[18]
710	710A	MaCYP710A1 (giant chickweed)	up-regulated in NTSR plants compared to sensitive plants	Sulfonylurea	1	[6]

References

1. Gardin JAC, Gouzy J, Carrère S, Délye C. ALOMYbase, a resource to investigate non-target-site-based resistance to herbicides inhibiting acetolactate-synthase (ALS) in the major grass weed *Alopecurus myosuroides* (black-grass). BMC Genomics. 2015;16(590).

2. Piasecki C, Yang Y, Benemann DP, Kremer FS, Galli V, Millwood RJ, et al. Transcriptomic analysis identifies new non-target site glyphosate-resistance genes in *Conyza bonariensis*. Plants. 2019;8(6):157.

3. Liu XM, Xu X, Li BH, Yao XX, Zhang HH, Wang GQ, et al. Genomic and transcriptomic insights into cytochrome P450 monooxygenase genes involved in nicosulfuron tolerance in maize (*Zea mays* L.). J Integr Agric. 2018;17(8):1790–9.

4. Bai S, Zhao Y, Zhou Y, Wang M, Li Y, Luo X, et al. Identification and expression of main genes involved in non-target site resistance mechanisms to fenoxaprop-p-ethyl in *Beckmannia syzigachne*. Pest Manag Sci. 2020;76(8):2619–26.

5. Iwakami S, Uchino A, Kataoka Y, Shibaike H, Watanabe H, Inamura T. Cytochrome P450 genes induced by bispyribac-sodium treatment in a multiple-herbicide-resistant biotype of *Echinochloa phyllopogon*. Pest Manag Sci. 2014;70(4):549–58.

6. Bai S, Liu W, Wang H, Zhao N, Jia S, Zou N, et al. Enhanced herbicide metabolism and metabolic resistance genes identified in tribenuron-methyl resistant *Myosoton aquaticum* L. J Agric Food Chem. 2018;66:9850–7.

7. Liu W, Bai S, Zhao N, Jia S, Li W, Zhang L, et al. Non-target site-based resistance to tribenuron-methyl and essential involved genes in *Myosoton aquaticum* (L.). BMC Plant Biol. 2018;18(1):225.

8. Franco-Ortega S, Goldberg-Cavalleri A, Walker A, Brazier-Hicks M, Onkokesung N, Edwards R. Non-target site herbicide resistance is conferred by two distinct mechanisms in black-grass (*Alopecurus myosuroides*). Front Plant Sci. 2021;12:636652.

9. Duhoux A, Carrère S, Duhoux A, Délye C. Transcriptional markers enable identification of rye-grass (*Lolium* sp.) plants with non-target-site-based resistance to herbicides inhibiting acetolactate-synthase. Plant Sci. 2017;257:22–36.

10. Leslie T, Baucom RS. De novo assembly and annotation of the transcriptome of the agricultural weed *Ipomoea purpurea* uncovers gene expression changes associated with herbicide resistance. G3 Genes, Genomes, Genet. 2014;4(10):2035–47.

11. Gaines TA, Lorentz L, Figge A, Herrmann J, Maiwald F, Ott M-C, et al. RNA-Seq transcriptome analysis to identify genes involved in metabolism-based diclofop resistance in *Lolium rigidum*. Plant J. 2014;78(5):865–76.

12. Salas-Perez RA, Saski CA, Noorai RE, Srivastava SK, Lawton-Rauh AL, Nichols RL, et al. RNA-Seq transcriptome analysis of *Amaranthus palmeri* with differential tolerance to glufosinate herbicide. PLoS One. 2018;13:1–33.

13. Matzrafi M, Shaar-Moshe L, Rubin B, Peleg Z. Unraveling the transcriptional basis of temperature-dependent pinoxaden resistance in *Brachypodium hybridum*. Front Plant Sci. 2017;8:1064.

14. Thyssen GN, Naoumkina M, McCarty JC, Jenkins JN, Florane C, Li P, et al. The P450 gene CYP749A16 is required for tolerance to the sulfonylurea herbicide trifloxysulfuron sodium in cotton (*Gossypium hirsutum* L.). BMC Plant Biol. 2018;18(1):186.

15. Pan L, Gao H, Xia W, Zhang T, Dong L. Establishing a herbicide-metabolizing enzyme library in *Beckmannia syzigachne* to identify genes associated with metabolic resistance. J Exp Bot. 2016;67(6):1745–57.

16. Wang J, Chen J, Li X, Cui H. RNA-Seq transcriptome analysis to identify candidate genes involved in non-target site-based mesosulfuron-methyl resistance in *Beckmannia syzigachne*. Pestic Biochem Physiol. 2021;171:104738.

17. Zhao N, Li W, Bai S, Guo W, Yuan G, Wang F, et al. Transcriptome profiling to identify genes involved in mesosulfuron-methyl resistance in *Alopecurus aequalis*. Front Plant Sci. 2017;8:1391.

18. Yang Q, Deng W, Li X, Yu Q, Bai L, Zheng M. Target-site and non-target-site based resistance to the herbicide tribenuron-methyl in flixweed (*Descurainia sophia* L.). BMC Genomics. 2016;17(1):17:551.
