# Supplementary material for: Genes encoding cytochrome P450 monooxygenases and glutathione S-transferases associated with herbicide resistance evolved before the origin of land plants
Source: PLoS One. 2023 Feb 17;18(2):e0273594. doi: 10.1371/journal.pone.0273594 (PMC9937507; doi:10.1371/journal.pone.0273594)
Supplement: S4 Table — (PDF) [file pone.0273594.s008.pdf]

S4 Table. Candidate NTSR GSTs belong to several GST classes.

Class	Gene name	Evidence	Herbicide chemical class	Genes per clan	References
DHAR	MaDHAR2 (giant chickweed)	Up-regulated in NTSR plants compared to sensitive plants	Sulfonylurea	2	[1]
	TtDHAR (wheat)	Induced by safener treatment			[2]
Lambda	TaGSTL (wheat)	Up-regulated in safener-treated plants compared to untreated plants	FOP, Sulfonylurea	2	[3]
	TtGSTL (wheat)	Induced by safener treatment			[2]
MAPEG	MaGSTM1 (giant chickweed)	Up-regulated in NTSR plants compared to sensitive plants	Sulfonylurea	1	[1]
Phi	BsGSTF1 (American slough grass)	Up-regulated in NTSR plants compared to sensitive plants	FOP	18	[4]
	MaGSTF11 (giant chickweed)	Up-regulated in NTSR plants compared to sensitive plants	Sulfonylurea		[1]
	ZmGSTI (maize)	Up-regulated in NTSR plants compared to sensitive plants	α-Chloroacetamide		[5]
	ZmGSTII (maize)	Induced by safener treatment, up-regulated in NTSR plants compared to sensitive plants	α-Chloroacetamide		[5,6]
	ZmGSTIII (maize)	Up-regulated in NTSR plants compared to sensitive plants	α-Chloroacetamide		[5,6]
	AmGSTF1 (blackgrass)	Higher glutathione peroxidase activity and higher protein levels in NTSR plants compared to sensitive plants	α-Chloroacetamide, DIM, Dinitroaniline, FOP, Phenylurea, Paraquat, Triazine		[7,8]
	AmGSTF2 (blackgrass)	Higher protein levels in NTSR plants compared to sensitive plants	α-Chloroacetamide, DIM, Dinitroaniline, FOP, Phenylurea, Paraquat, Triazine		[7]
	OsGSTII (rice)	Up-regulated in NTSR plants compared to sensitive plants	α-Chloroacetamide		[9]
	TaGSTF I-II (wheat)	Up-regulated in safener-treated plants	FOP, Sulfonylurea		[3]
	LrGSTF1 (annual rye-grass)	Enhanced protein levels in NTSR plants compared to sensitive plants	Glycine, Sulfonylurea, FOP		[10]
	LrGSTFA (annual rye-grass)	Up-regulated in NTSR plants compared to sensitive plants	Sulfonylurea		[11]
	Contig16302 (annual rye-grass)	Up-regulated in NTSR plants compared to sensitive plants	FOP		[12]
	SbGSTF1 (sorghum)	Up-regulated in safener-treated plants			[13]
	SbGSTF2 (sorghum)	Up-regulated in safener-treated plants			[13]
	AtuGSTF2 (waterhemp)	Up-regulated in NTSR plants compared to sensitive plants			[14]
	TtGSTF1 (wheat)	Induced by safener treatment			[2]
	TtGSTF2 (wheat)	Induced by safener treatment			[2]
	TtGSTF3 (wheat)	Induced by safener treatment			[2]
	TtGSTF4 (wheat)	Induced by safener treatment			[2]
	TtGSTF5 (wheat)	Induced by safener treatment			[2]
	TtGSTF6 (wheat)	Induced by safener treatment			[2]
Tau	ZmGST5 (maize)	Up-regulated in NTSR plants compared to sensitive plants	α-Chloroacetamide	22	[15]
	ZmGST6 (maize)	Up-regulated in NTSR plants compared to sensitive plants	α-Chloroacetamide		[15]
	ZmGST7 (maize)	Up-regulated in NTSR plants compared to sensitive plants	α-Chloroacetamide		[15]
	EcGST1 (barnyard grass)	Up-regulated in NTSR plants compared to sensitive plants	α-Chloroacetamide, Quinoline-carboxylate		[16]
	OsGSTI (rice)	Up-regulated in NTSR plants compared to sensitive plants	α-Chloroacetamide		[9]
	TaGSTUI-I (wheat)	Up-regulated in safener-treated plants	FOP, Sulfonylurea		[3]
	AmGSTU2 (blackgrass)	Higher protein levels in NTSR plants compared to sensitive plants	α-Chloroacetamide, DIM, Dinitroaniline, FOP, Phenylurea, Paraquat, Triazine		[7]
	LrGSTUI (rye-grass)	Up-regulated in NTSR plants compared to sensitive plants	Sulfonylurea		[11]
	LrGSTUJ (rye-grass)	Up-regulated in NTSR plants compared to sensitive plants	Sulfonylurea		[11]
	LrGSTUK (rye-grass)	Up-regulated in NTSR plants compared to sensitive plants	Sulfonylurea		[11]
	Contig5390 (rye-grass)	Up-regulated in NTSR plants compared to sensitive plants	FOP		[12]
	Contig13326 (rye-grass)	Up-regulated in NTSR plants compared to sensitive plants	FOP		[12]

S4 Table. Candidate NTSR GSTs belong to several GST classes (continued).

Class	Gene name	Evidence	Herbicide chemical class	Genes per clan	References
	BsGSTU1 (American slough grass)	Up-regulated in NTSR plants compared to sensitive plants	FOP		[17]
	BsGSTU3 (American slough grass)	Up-regulated in NTSR plants compared to sensitive plants	FOP		[17]
	TtGSTU1 (wheat)	Induced by safener treatment	α-Chloroacetamides		[2,18–20]
	TtGSTU2 (wheat)	Induced by safener treatment	α-Chloroacetamides		[2,19,20]
	TtGSTU3 (wheat)	Induced by safener treatment	α-Chloroacetamides		[2,20]
	TtGSTU4 (wheat)	Induced by safener treatment	α-Chloroacetamides		[2,20]
	TtGSTU5 (wheat)	Induced by safener treatment	α-Chloroacetamides		[2,20]
	TtGSTU6 (wheat)	Induced by safener treatment	α-Chloroacetamides		[2]
	TtGSTU7 (wheat)	Induced by safener treatment	α-Chloroacetamides		[2]
	TtGSTU8 (wheat)	Induced by safener treatment	α-Chloroacetamides		[2]
Theta	BsGSTT3 (American slough grass)	Up-regulated in NTSR plants compared to sensitive plants	FOP	1	[17]

References

1. Bai S, Liu W, Wang H, Zhao N, Jia S, Zou N, et al. Enhanced herbicide metabolism and metabolic resistance genes identified in tribenuron-methyl resistant *Myosoton aquaticum* L. J Agric Food Chem. 2018;66:9850–7.

2. Zhang Q, Riechers DE. Proteomic characterization of herbicide safener-induced proteins in the coleoptile of *Triticum tauschii* seedlings. Proteomics. 2004;4(7):2058–71.

3. Taylor VL, Cummins I, Brazier-Hicks M, Edwards R. Protective responses induced by herbicide safeners in wheat. Environ Exp Bot. 2013;88:93–9.

4. Bai S, Zhao Y, Zhou Y, Wang M, Li Y, Luo X, et al. Identification and expression of main genes involved in non-target site resistance mechanisms to fenoxaprop-p-ethyl in *Beckmannia syzigachne*. Pest Manag Sci. 2020;76(8):2619–26.

5. Li D, Gao Q, Xu L, Pang S, Liu Z, Wang C, et al. Characterization of glutathione S-transferases in the detoxification of metolachlor in two maize cultivars of differing herbicide tolerance. Pestic Biochem Physiol. 2016;143:265–71.

6. Fuerst EP, Irzyk GP, Miller KD, Townson J, Edwards R. Partial characterization of glutathione S-transferase isozymes induced by the herbicide safener benoxacor in maize. Plant Physiol. 1993;102(3):795–802.

7. Tétard-Jones C, Sabbadin F, Moss S, Hull R, Neve P, Edwards R. Changes in the proteome of the problem weed blackgrass correlating with multiple-herbicide resistance. Plant J. 2018;94(4):709–20.

8. Franco-Ortega S, Goldberg-Cavalleri A, Walker A, Brazier-Hicks M, Onkokesung N, Edwards R. Non-target site herbicide resistance is conferred by two distinct mechanisms in black-grass (*Alopecurus myosuroides*). Front Plant Sci. 2021;12:636652.

9. Wu J, Cramer CL, Hatzios KK. Characterization of two cDNAs encoding glutathione *S*- transferases in rice and induction of their transcripts by the herbicide safener fenclorim. Physiol Plant. 2002;105(1):102–8.

10. Cummins I, Wortley DJ, Sabbadin F, He Z, Coxon CR, Straker HE, et al. Key role for a glutathione transferase in multiple-herbicide resistance in grass weeds. Proc Natl Acad Sci. 2013;110(15):5812–7.

11. Duhoux A, Carrère S, Duhoux A, Délye C. Transcriptional markers enable identification of rye-grass (*Lolium* sp.) plants with non-target-site-based resistance to herbicides inhibiting acetolactate-synthase. Plant Sci. 2017;257:22–36.

12. Gaines TA, Lorentz L, Figge A, Herrmann J, Maiwald F, Ott M-C, et al. RNA-Seq transcriptome analysis to identify genes involved in metabolism-based diclofop resistance in *Lolium rigidum*. Plant J. 2014;78(5):865–76.

13. Baek YS, Goodrich L V., Brown PJ, James BT, Moose SP, Lambert KN, et al. Transcriptome profiling and genome-wide association studies reveal GSTs and other defense genes involved in multiple signaling pathways induced by

herbicide safener in grain sorghum. Front Plant Sci. 2019;10:192.

14. Evans AF, O'Brien SR, Ma R, Hager AG, Riggins CW, Lambert KN, et al. Biochemical characterization of metabolism-based atrazine resistance in *Amaranthus tuberculatus* and identification of an expressed GST associated with resistance. Plant Biotechnol J. 2017;15(10):1238–49.

15. Li D, Xu L, Pang S, Liu Z, Wang K, Wang C. Variable levels of glutathione S-transferases are responsible for the differential tolerance to metolachlor between Maize (*Zea mays*) shoots & roots. J Agric Food Chem. 2017;65(1):39–44.

16. Li G, Wu SG, Yu RX, Cang T, Chen LP, Zhao XP, et al. Identification and expression pattern of a glutathione S-transferase in *Echinochloa crus-galli*. Zwerger P, editor. Weed Res. 2013;53(5):314–21.

17. Pan L, Gao H, Xia W, Zhang T, Dong L. Establishing a herbicide-metabolizing enzyme library in *Beckmannia syzigachne* to identify genes associated with metabolic resistance. J Exp Bot. 2016;67(6):1745–57.

18. Riechers DE, Irzyk GP, Jones SS, Fuerst EP. Partial characterization of glutathione S-transferases from wheat (*Triticum* spp.) and purification of a safener-induced glutathione S-transferase from <i>Triticum tauschii. Plant Physiol. 1997;114(4):1461–70.

19. Xu F, Lagudah ES, Moose SP, Riechers DE. Tandemly duplicated safener-induced glutathione S-transferase genes from *Triticum tauschii* contribute to genome- and organ-specific expression in hexaploid wheat. Plant Physiol. 2002;130(1):362–73.

20. Zhang Q, Xu F, Lambert KN, Riechers DE. Safeners coordinately induce the expression of multiple proteins and MRP transcripts involved in herbicide metabolism and detoxification in *Triticum tauschii* seedling tissues. Proteomics. 2007;7(8):1261–78.
