# Supplementary material for: Genes encoding cytochrome P450 monooxygenases and glutathione S-transferases associated with herbicide resistance evolved before the origin of land plants
Source: PLoS One. 2023 Feb 17;18(2):e0273594. doi: 10.1371/journal.pone.0273594 (PMC9937507; doi:10.1371/journal.pone.0273594)
Supplement: S5 Table — Sequences from these classes were not included in the phylogenetic analysis because they lack the classical N-terminal and C-terminal GST domains. 2N GST sequences have two N-terminal domains and lack a C-terminal domain. Kappa GST proteins lack both N and C-terminal GST domains and instead have a single thioredoxin-like kappa GST domain (InterPro domain IPR014440). MAPEG GST proteins lack both C and N-terminal GST domains and have instead a single ‘MAPEG’ domain (InterPro domain IPR001129). (PDF) [file pone.0273594.s009.pdf]

S5 Table. Number of GST proteins identified from classes 2N, Kappa, and MAPEG in green plants and red algae.

Species	2N	Kappa	MAPEG
Arabidopsis thaliana	2	0	1
Oryza sativa	1	0	1
Selaginella moellendorffii	2	0	2
Anthoceros agrestis	1	0	2
Physcomitrium patens	1	1	4
Marchantia polymorpha	2	1	2
Klebsormidium nitens	3	1	1
Chlamydomonas reinhardtii	1	0	1
Cyanidioschyzon merolae	1	0	1

Sequences from these classes were not included in the phylogenetic analysis because they lack the classical N-terminal and C-terminal GST domains. 2N GST sequences have two N-terminal domains and lack a C-terminal domain. Kappa GST proteins lack both N and C-terminal GST domains and instead have a single thioredoxin-like kappa GST domain (InterPro domain IPR014440). MAPEG GST proteins lack both C and N-terminal GST domains and have instead a single 'MAPEG' domain (InterPro domain IPR001129).
