# Supplementary material for: Genes encoding cytochrome P450 monooxygenases and glutathione S-transferases associated with herbicide resistance evolved before the origin of land plants
Source: PLoS One. 2023 Feb 17;18(2):e0273594. doi: 10.1371/journal.pone.0273594 (PMC9937507; doi:10.1371/journal.pone.0273594)
Supplement: Attachment — Submitted filename: PONE-D-22-22418_Casey_etal_response to reviewers.docx [file pone.0273594.s020.docx]

**Responses to the referees**

We are extremely grateful for the detailed, constructive and insightful comments of each of the three referees. We are also grateful for the errors that they identified in the previous version. Their recommendations have allowed us to improve the quality of our manuscript. Thank you.

**Reviewer #1: General Comments:**
This manuscript is well-written, clear, and organized. The topic offers a new perspective on herbicide resistance making the subject of interest to weed scientists as well as those interested in molecular phylogenetics. The article is a unique blend a literature review of NTS-based herbicide resistance and the role specific CYPs and GSTs play in that resistance.

Specific Comments/Questions:
Throughout the manuscript there is mention of hypotheses and how the data support various hypotheses (lines 266, 271, 368, 378, 390, 486, 489, 507). This hypothesis-testing emphasis allows the reader to read the document with the purpose in mind. However, it is unclear as to whether the hypotheses mentioned are the authors’ or the disciplines. In my opinion, I would state the hypotheses being addressed and tested up front in the introduction so that the reader can read the article with a goal in mind.

Response:

We understand the ambiguity in our text identified by the referee. We have rewritten the highlighted sections to clarify that these are the authors’ hypotheses.

One improvement to the document would be to add more information on the phylogeny of the species selected for the analysis. Table 1 partially serves this purpose. Maybe Table 1 could be modified to include how these vascular and non-vascular plants and alga fit into the kingdom. Also, terms like embryophyte, bryophyte, glaucophyte, and Viridiplante are used without context or definition. Adding this information would make the manuscript an easier read for agronomists and weed scientists.

Response:

We have made the changes recommended by the referee in Table 1 and in the text.

Another piece of information that would be helpful is the rationale used to determine which species to include in this analysis. First, why were these classes selected? For example, are ferns and conifers included? Also, of the 9 species selected for the analysis, why these? Is it due to genomic information or genome size? Are they recognized representatives of their classes in the field of plant phylogeny?

Response:

We included rationales in the new version.

The authors imply that TSR and NTSR of weeds is due to mutations (first paragraph of introduction; Lines 462-463; Line 524). This is not always true. For example, could an herbicide molecule and a native substrate for a CYP or GST both fit in the enzyme’s active site? Also, some NTSR cases of weed resistance may be due to the accumulation of alleles that, when expressed, together confer tolerance to an herbicide (i.e., multigenic). If the term mutation is being more broadly used to include gene duplication or changes in gene regulation, it should be made clear.

Response:

The reviewer makes very good points. We have modified the text as suggested.

Finally, from the literature search and data analyses conducted, are the authors able to determine what percentage of NTSR cases are due to CYPs and/or GSTs? What percentage of metabolism-based resistance cases are not linked to any genes (unaccounted for)? Are there any linkages with the chemical classes subject to metabolism by GSTs and CYPs? In other words, are there any chemical motifs susceptible to metabolism by CYPs and GSTs? Determining the native function of these enzymes might enable this linkage.

Response:

We are unaware of any chemical motifs that are more susceptible to metabolism by GSTs or CYPs, as suggested by the reviewer. We mention this in the Discussion, to make this clear to the reader.

Minor Suggestions/Corrections:
Introduction
Line 44: Does “inhibit the interaction between the two” mean “reduce affinity of the herbicide for the target site”?

Response:

Yes it does, we modified the revised text to reflect this.

Line 72: Is there a description for Ure2p?

Response:

Yes, we have included it in the revised text.

Lines 70-74: Is there a reference for this statement/lists?

Response:

Yes, we have included them in this section.

Line 93: A brief definition of Archaeplastida would be helpful here.

Response:

This has been included in the next paragraph of the revised text.

Line 96: It would be helpful to define MYA for its first use.

Response:

This has been added to the revised text.

Line 99: Since this is the first mention of K. nitens in the document, the genus name should be used.

Response:

We included this change in the revised text.

Materials & Methods

Line 116: Since this is the first mention of A. thaliana in the document, the genus name should be used. Also need a period at end of sentence.

Response:

This has been corrected in the revised text.

Line 117: Was the ssp. japonica sequence used for Oryza sativa? If so, please indicate here. Figure 1 legend indicates the japonica sequence was used.

Response:

Yes, this information was added to the revised text.

Line 118: Since this is the first mention of M. polymorpha in the document, the genus name should be used.

Response:

This has been corrected in the revised version.

Lines 116-127: Why is the protein sequence reference for S. moellendorffii not included with the other?

Response:

This information has been added to the revised text.

Line 146: Maybe change “don’t” to “do not”.

Response:

This has been changed in the revised text.

Results
Line 255: Define Viridiplantae here if not added earlier in the manuscript.

Response:

This has been added to the revised text and to Table 1.

Line 280: Phrase “of the” repeated in sentence.

Response:

This has been removed from the revised.

Line 283: For consistency use MYA?

Response:

This has been modified in the revised text.

Line 284: Reference fig 2C?

Response:

We have checked and corrected all references to fig 2C.

Line 284: Should this be four CYPs instead of 5 CYPs?

Response:

Yes, this has been corrected in the revised text.

Line 303: Start sentence with “Sixteen”.

Response:

This has been corrected in the revised text.

Line 323: Iota is shown in the Rhodophytes

Response:

Yes, this manuscript identifies an Iota GST in *C. merolae*, indicating that Iota GSTs predate the divergence of rhodophytes and chlorophytes.

Line 347: Start sentence with “Twelve”.

Response:

This has been corrected in the revised text.

Lines 348-350: Is there a reference for this statement? There is no information in the manuscript to differentiate these 4 classes from the other 7 that also are shown as the earliest.

Response:

I have included the missing information and references in this section of the revised text.

Lines 399-401: How can these data account for there being only 1 Phi GST in S. moellendorffii? Please address.

Response:

This has now been addressed in the paragraph of the revised text.

Lines 427 & 448: “30” instead of “thirty”.

Response:

This has been changed in the revised text.

Lines 435 & 436: It appears the 29 should be 30.

Response:

That is correct, this was corrected in the revised text.

Lines 449-450: It would be useful to define what chemical classes FOP, DIM, and DEN represent.

Response:

This information has been included in the revised text.

Lines 454-455: Sentence is not complete.

Response:

This is corrected in the revised text.

Line 481: How is the 50-70% estimate derived?

Response:

This estimate was derived from previously published GST phylogenies, however it has been amended to refer to the species in this paper.

Discussion
Line 501: How were the glaucophyte alga represented?

Response:

One Glaucophyte species nuclear genome has been sequenced to date, (*Cyanophora paradoxa)*. It was an oversight not to include it.

Line 503: Should the 71 be 710?

Response:

Yes, that is correct, and has been corrected in the revised text.

Lines 508-509: Statement about gene duplications needs a reference.

Response:

References 1 and 2 have been cited.

Pégeot H, Koh CS, Petre B, Mathiot S, Duplessis S, Hecker A, et al. The poplar Phi class glutathione transferase: expression, activity and structure of GSTF1. Front Plant Sci. 2014;5:712.

Werck-Reichhart D, Feyereisen R. Cytochromes P450: a success story. Genome Biol. 2000;1(6):1–9.

Line 522: Supplemental tables indicate crop plants also are resistant due to CYP clans 71 and 72 and GST classes Tau and Phi.

Response:

Yes, this has been added to the revised text.

Lines 536-541: Are references needed for the statements concerning the different eras mentioned?

Response:

Reference 131 has been inserted.

Cohen KM, Finney SC, Gibbard PL, Fan J-X. ICS International Chronostratigraphic Chart 2022/10 [Internet]. International Commission on Stratigraphy, IUGS. 2022 [cited 2022 Oct 16]. Available from: www.stratigraphy.org

Figures/Tables
Figures 1-2: The colors are not clear/distinct; could this be improved?

Response:

Yes, the colours and figures have been made clearer in Figures 1 and 2.

Figure 2, line 262: Legend states 4 CYP clans are shown, but the figure shows 5. Maybe state increases in 4 of the 5 shown.

Response:

The figure legend has been changed accordingly in the revised text.

**Reviewer #2:** The manuscript describes the phylogenetic analysis of two types of enzymes that have key roles in non-target resistance to weeds from an evolutionary standpoint. The phylogenetic analysis appears to be very rigorous, but a slight weakness of the paper is around the descriptions of the basis of NTSR which are unclear in places.

Abstract

Line 32 ‘chemically alter’ is better described as ‘metabolism’ with these enzymes. This phrasing is repeated several times within the manuscript.

Response:

Chemically alter has been replaced with metabolise throughout the revised text.

Introduction

Lines 46-52. The three sentences could do with redrafting as it comes across as unclear and repetitive.

Response:

They have been rewritten more concisely.

Line 51 what is meant by ‘hyperactive forms of the enzymes’. I haven’t come across this terminology before.

Response:

We have removed “hyperactive forms of the enzymes” from the text. This was included in error and we are grateful to the referee for spotting this.

Line 52 Ref. 5 is not a suitable reference in this context as it is an example of forced evolution in the laboratory. Are there any examples of enzymes with altered substrate specificity arising in nature?

Response:

This reference has been removed.

Line 93 sentence should be amended to ‘CYPs and GSTs discovered so far that confer herbicide resistance.

Response:

This sentence has been amended as requested.

Results

Table 1. Classification of Arabidopsis referred to as eudicot in table 1 but as a dicot in fig. 1. Would make comprehension easier if were consistent.

Response:

This has been amended in the revised text.

Fig.1 The key refers to the classification but in the text the actual species are discussed. This makes it difficult to understand unless you are very familiar with the classification categories. Either both or species should be added to the figure key.

Response:

The key has been modified to include species names in Figure 1.

Fig. 2 need to add units to the graph.

Response:

Units have been added to the revised Figure 2.

Line 322 Definition of ‘Archaeplastida’ would be useful for the more general reader.

Response:

A definition of Archaeplastida has been included in the Introduction and in Table 1.

Line 443 should refer to class for GSTs not clan

Response:

This has been corrected.

Discussion

Lines 524 -529. The enzymatic activity of these proteins is surely the most important reason as to why these clans and classes are important. Other CYPs and GSTs are up-regulated in NTSR populations but without activity towards herbicides are unlikely to have a major standalone effect on resistance.

Response:

Yes, this is a good point. However, it is unclear precisely what enzymatic activity unites the enzymes in these classes. This has now been included in the text between lines 537 and 555.

Funding

Line 557 lacks a grant number.

Response:

This has been amended.

Supporting information

Table S3 Why are the 81As highlighted?

 Response:

This was in error and the highlighting has been removed.

**Reviewer #3:** Genes encoding cytochrome P450 monooxygenases and glutathione S-transferases associated with herbicide resistance evolved before the origin of land plants

General Summary

The authors used publicly available genomic data to perform a phylogenetic analysis to uncover the origins of GST and CYPs in land plants. The discovered that certain clans within these enzymes were especially abundant in land plants while others were completely lost indicating that the expansion of these metabolic enzyme clans has a strong advantage for land plants. Furthermore, these expansion may help explain land plant evolution of non-target site resistance

General Comments

The writing was excellent. Very clear and thorough analysis. My biggest criticisms include
1) the results being redundant with much of the methods. Results can be tighter and reduced for readability.

Response:

We have deleted redundant text. The Results of the revised manuscript are more concise than the original version.

2) Needs a better discussion of the Native functions of CYPs, from before herbicides were available. Land plant evolved much before agriculture so what drove certain clan expansion while others atrophied.

Response:

This is an important point and has been addressed in the Discussion (lines 535 to 555 and 563 to 570).

3) I think that perhaps some of the supplementary figures/tables, are quite important to understanding the discussion more completely. For instance, For me, Table S3 was valuable.

Response:

This a good point, and Table S3 and Table S4 have been included in the paper as Table 2 and Table 3.

Line Comments
Ln 33: Please indicate briefly here that Cyp81s fall under the cyp 71 clan. Due to the importance of CYP81s in NTSR, this will be critical information to have upfront in the abstract for people who don’t read any further.

Response:

This information has been included.

Ln 45-49: These sentences are largely redundant and can be condensed into a single statement.

Response:

This section has been rewritten to be more concise.

Ln 49: I would imagine that the SNPs are not necessarily in the genes themselves, especially if they are changing the expression of a NTSR gene. Mutations in the gene body might modify NTSR protein ligand affinity, however, It would be more accurate to say genetic changes in the promoter of NTSR genes and/or the promoters of transcription factors that then in turn regulate NTSR gene expression are responsible for changing gene expression.

Response:

This is a very good point and the text has been modified to reflect this.

Ln 56: ‘Has’ should be ‘Have’

Response:

Overexpression is a singular noun and should be associated with a singular verb (has). “The overexpression of … has ….” therefore we have not changed “has” to “have” in this case.

Ln 97-101: Missing in this hypothesis is the initial reason land plants would have more of these specific CYPs/GSTs. Surely clan number increase occurred well before the advent of herbicides. What evolutionary benefit would having more CYP/GST diversity in the clans provide in the absence of herbicides and why is it different for vascular plants then Archaeplastida.

Response:

This is an interesting point. We suspect that it is related to the increase in chemical and physiological diversity, but there is no evidence to support it. Therefore, we have not added a discussion of this point to the final sentence of the closing paragraph of the introduction. However, we added text that addresses this point at lines 563 to 565 of the Discussion.

*“This expansion likely accompanied an increase in diversity of signalling molecules and secondary metabolites that may have occurred soon after plants started to grow in relatively dry terrestrial environments.”*

Ln 155-156: Can there a description somewhere of what those ‘important residues’ might be? As is I, with my limited knowledge of cyp protein sequence, could not replicate your alignments and therefor your results.

Response:

Yes, this is illustrated in supplementary figures S1, S3, and S4 however this wasn’t stated in the text. A sentence has now been included at this section referring to these figures.

Ln 213-223: There is a lot of re-hashing of methods in the results. It makes the results bloated. I suggest being more brief.

Response:

This paragraph has been moved to Methods.

Ln 247-250: Is it possible that these 6 clans (71, 72, 74, 85, 86, and 727) evolved before the split of chlorophytes and streptophytes but were lost due to lack of selection? The divergence happen so long ago I could imagine lots of gene loss. I don’t know the phylogeny of land plants well enough to defend this hypothesis, I am curious if you have a reason to not favor this hypothesis.

Response:

It is possible but unlikely because these 6 clans were not present in the genomes of both *C. reinhardtii* and *C. merolae*. Therefore, the most parsimonious scenario is that these clans evolved in the last common ancestor of streptophytes.

Ln448-464: When mentioning the chemical classes it may be valuable for some readers to indicate what enzyme these chemistries inhibit or classify them by their HRAC code (Herbicide resistance Action Committee). Many weed scientists think about chemistries grouped like this.

Response:

This is a good point, HRAC codes have been included for each chemical class.

Ln494-511: I would appreciate a discussion of native Cyp and GST function. The diversification of these clades in land plants was not driven by herbicides as it happened well before human agriculture. Maybe these clans are particularly useful in pathogen defense, antiherbivory, or chemical defense. Also, are plants with more CYPs in these clans more likely to evolve resistance? Could that be hypothesized and tested using similar phylogenetic approaches?

Response:

This is a good point and we have inserted a paragraph between lines 535 and 555 that addresses this issue. However, there is little known and therefore our discussion is very speculative.
