# Supplementary material for: Genes encoding cytochrome P450 monooxygenases and glutathione S-transferases associated with herbicide resistance evolved before the origin of land plants
Source: PLoS One. 2023 Feb 17;18(2):e0273594. doi: 10.1371/journal.pone.0273594 (PMC9937507; doi:10.1371/journal.pone.0273594)
Supplement: Attachment — Submitted filename: 20230125_Response_to_reviewers.docx [file pone.0273594.s021.docx]

**Response to reviewers and editor:**

We are extremely grateful for the detailed comments and concerns noted by the reviewers as they have further improved the quality of the manuscript and our understanding of the field. Please find below our responses to each of the comments in turn.

Please review your reference list to ensure that it is complete and correct. If you have cited papers that have been retracted, please include the rationale for doing so in the manuscript text, or remove these references and replace them with relevant current references. Any changes to the reference list should be mentioned in the rebuttal letter that accompanies your revised manuscript. If you need to cite a retracted article, indicate the article’s retracted status in the References list and also include a citation and full reference for the retraction notice.

Additional Editor Comments:

**Academic Editor's synthesis comments.** Thank you for submitting your revised manuscript and detailed responses to the first three reviewers’ comments. One of the three original reviewers has again read and critiqued your revised manuscript and their comments are listed below. In addition, I have also read your original and revised manuscript thoroughly and have detailed below several additional comments and minor concerns that I’d like you to address in the new revised manuscript. If you can satisfactorily respond to and address these comments in your revision then I should be able to provide a quick turnaround. 

An important concern that should be fairly straightforward to address (without additional analysis) is that you are not using the terms herbicide tolerance and herbicide resistance correctly, per the Weed Science Society of America definitions of tolerance and resistance (see wssa.net for more information). Sometime you mention tolerance and resistance in the same sentence (e.g**., lines 23-25 and 530-531**) and sometimes you mention resistance when you actually should be stating tolerance. The WSSA definitions are as follows: (**a**) tolerance refers to a plant species that is not controlled at a herbicide rate that kills other species; tolerance is therefore considered “natural” and does not imply any type of selection pressure or artificial genetic manipulation, and (**b**) resistance refers to a plant or population of a species that is not controlled by a herbicide rate that typically kills plants within this same species; resistance therefore implies selection by a herbicide (or other factor) or artificial genetic manipulation, such as transgenic methods to make a GM crop variety. 

Response: we are grateful to the editor for pointing this out to us, and agree these terms should be used correctly and consistently. The recommended changes have been made to the manuscript.

In many cases you wrote resistance when you are actually referring to a naturally tolerant crop or weed species. Examples of this are the majority of the crop species included in your CYP and GST analyses; these detoxification enzymes confer natural (or safener-induced) tolerance, and is therefore not involved with resistance and did not result from any known selection pressure. I do not believe this issue with your manuscript text is a deal breaker by any means, but I do believe you need to clearly rewrite portions of the manuscript and several table headings and figure captions. I realize that it would be much easier to include all CYPs and GSTs in your analysis under the “resistance” category, but unfortunately this is not scientifically accurate and could be misleading.

Response: we thank the editor for this comment and completely agree. The recommended changes have been made to the text, table headings and figure captions.

Other comments:

Line 147; reword “self-blasted” with a scientific term.

Response: the sentence has been reworded.

Lines 194-209; just an optional idea, but would it be informative to include the average and/or median number of GST and CYP genes in all populations you examined for comparison? If you disagree then that is fine, but please explain in your rebuttal comments.

Response: The average number of CYP genes identified in the 9 species is 126 sequences per species. The average number of GST genes identified in the 9 species is 40 sequences per species. This has been included in the paragraph.

Lines 384-86 and 407-409; I understand what you mean by writing about increases in CYPs and GSTs in certain species arising from specific clans or classes, but instead of “increase” would it be more accurate to say “enrichment” or another similar term? In other words, are you saying that the total number of protein-coding genes among species is about the same, but the number of CYPs and GSTs increased disproportionately? Maybe enrichment isn’t the best term either, but I think it might be worth explaining this concept in a bit more detail.

Response: That is indeed what we mean. The sections of text have been reworded and expanded to hopefully explain this more clearly.

Lines 434-442, Tables 2 and 3, Tables S3 and S4; I have several comments here to consider and address: (**a**) as I mentioned earlier, it is not completely accurate to include all of these CYPs and GSTs under the category of resistance, since many of these genes and enzymes were discovered in naturally herbicide-tolerant crops – you could possibly change the title or heading to address this;

Response: this has been changed in the text and headings.

(**b**) something that I believe would be interesting and important to discuss is the ratio of tau : phi GSTs in plant species where complete genomes are available. Several review articles have speculated about why the ratio of tau : phi GST varies among species (while total GSTs per genome also can vary slightly), but do you have any thoughts or insights on this varying ratio observation? For example, some reviews have speculated about different biochemical functions for tau vs phi plant GSTs;

Response: this is a very interesting point. The ratio of Tau to Phi GSTs varies a lot between species and may be related to different biochemical functions fulfilling different environmental and ecological needs. This observation has been included in the results section in a new paragraph in lines 418 to 426 and in the discussion section in lines 547 to 549.

and (**c**) were your lists of GSTs and CYPs (regular in-text Tables and suppl. Tables for candidate genes) and references cited intended to be comprehensive via literature searches? I don’t normally like to do this as a journal peer reviewer or editor, but it seems unusual if you had comprehensively searched the literature that you would have missed several plant GST/CYP papers on tolerance/resistance published by my research group since the late 1990s. I will list these paper citations below for you to consider (either as verified detox gene/enzymes or candidates), and if you disagree with including them that is fine, but again please provide a justification in your rebuttal letter.

Baek, Y.S., Goodrich, L.V., et al. (2019). Front. Plant Sci. 10:192. doi:10.3389/fpls.2019.00192
Evans, A.F., O’Brien, S.R., et al. (2017). Plant Biotechnol. J. 15:1238-1249.
Pataky, J.K., Williams, M.M., et al. (2009). J. Amer. Soc. Hort. Sci. 134:252-260.
Nordby, J.N., Williams, M.M., et al. (2008). Weed Sci. 56:376-382.
Zhang, Q, Xu, F.-X., et al. (2007). Proteomics 7:1261-1278.
Zhang, Q., Riechers, D.E. (2004). Proteomics 4:2058-2071.
Xu, F.-X., Lagudah, E.S., et al. (2002). Plant Physiol. 130:362-373.
Riechers, D.E., Irzyk, G.P., et al. (1997). Plant Physiol. 114:1461-1470.

Response: thank you for pointing out this oversight. They should be in the tables and have been included.

Line 476 and Figure 3; using white, light gray and dark gray bars is not ideal for differentiating the columns (bars) presented. If you do not want to include color, then can you at least use hatch marks or some other distinguishing feature besides shades of gray?

Response: the colours of the bars in Figure 3 have been improved.

Line 478 and elsewhere in the manuscript; you briefly mention that CYP81 family members fall within the CYP71 clan, but I think you should clearly differentiate CYP clan vs family somewhere in the paper (perhaps even earlier in the introduction). The Hansen et al. 2021 CYP review has a nice box/figure that explains this topic, but I believe explaining this more in your paper will greatly help readers to understand the CYP nomenclature system…with can be confusing to expert and non-experts!

Response: this is a fair point and an explanation of CYP classification into clans and families has been included in the introduction.

The two small paragraphs in lines 496-502 are poorly worded and confusing. For example, “mutate to herbicide resistance” is poorly worded and doesn’t make sense scientifically. Please rewrite these sentences and ideally merge into one cohesive paragraph.

Response: these paragraphs have been rewritten and merged.

Lines 512 and 516; please include HRAC group 15 at first mention of chloroacetanilides in line 512.

Response: the HRAC group has been added.

Lines 522-534; several issues with these sentences: (**1**) Do not start a paragraph/sentence with a number (522), (**2**) as mentioned earlier, I think the ratio of tau : phi class GSTs among species may be important to discuss and speculate about in addition to total number of GSTs per genome, (**3**) I would not consider A. thaliana to be a herbicide-tolerant weed…it is sensitive to many herbicides that kill dicots (530-31), and (**4**) the final brief paragraph basically restates what you’ve already written and should either be expanded, deleted, or merged with the previous paragraph.

Response: All recommended changes have been made to this paragraph.

Lines 539-540; I agree with the reviewer that you cannot always assume resistance or tolerance is due to a change in GST or CYP expression. It is possible that amino acid changes in the protein could enhance herbicide substrate affinity and/or enzyme catalytic detox efficiency with herbicide substrates. Please reword to include alternative explanations.

Response: the sentence has been reworded.

Lines 540-41; as I mentioned earlier, resistance indeed implies changes resulting from selection pressures, but you cannot include natural crop or weed tolerance in this category.

Response: this sentence has been reworded.

Lines 554-55; you cannot mix (i.e., use interchangeably) resistance and tolerance in the same sentence.

Response: Tolerance has been replaced with resistance in this sentence.

Lines 561-562; as noted by the reviewers, a lot of your Discussion section is redundant with the Results section. It seems as though I’m reading the same sentences numerous times throughout the manuscript. Please reword, condense, or delete as needed.

Response: several sentences have been deleted.

Line 566; I agree with the reviewer about possibly rewording “mutation” in light of gene copy number variation, gene duplication, etc.

Response: this sentence has been reworded.

Lines 583-587; one unifying feature of GST substrates (natural or xenobiotic) is an electrophilic carbon that can be attacked by the thiolate anion of GS-; please see our review article below (Riechers et al. 2010), but several other papers on GSTs have also mentioned this possible unifying factor.

Riechers, D.E., Kreuz, K. and Zhang, Q. (2010). Plant Physiol. 153:3-13.

Response: This is a good point and has been included in the paragraph.

Lines 606-08: this concluding sentence seems too strong and all-encompassing; please reword or soften a bit. As the reviewer also pointed out, I do not believe we can always assume that differences in CYP or GST gene expression account for natural tolerance or evolved weed resistance; for example, please consider coding region changes that may alter herbicide substrate specificity, enzyme activity, or other enzymatic detox functions that may also play a large role (or gene duplication, Copy Number Variation, etc.). Even if examples have not yet been cited in the published literature, I think it is appropriate to speculate here.

Response: the sentence has been softened and the other possible changes that could alter herbicide resistance/tolerance have been included.

Optional comment to consider about genes/enzymes other than CYPs or GSTs that govern crop tolerance: I believe one of the original reviewers mentioned or asked something about genes other than CYPs/GSTs involved in tolerance or resistance. It is purely up to you, but I thought I’d list that paper below that describes a novel type of oxygenase (HIS1; Fe(II)/2-oxoglutarate-dependent oxygenase) in rice that was discovered due to varietal sensitivity issues to HPPD inhibitors (Group 27 herbicides). Maeda, H., Murata, K., et al. (2019) A rice gene that confers broad-spectrum resistance to β-triketone herbicides. Science 365:393-396.


[Note: HTML markup is below. Please do not edit.]

**Reviewers' comments:** 

Reviewer's Responses to Questions

**Comments to the Author**

1. If the authors have adequately addressed your comments raised in a previous round of review and you feel that this manuscript is now acceptable for publication, you may indicate that here to bypass the “Comments to the Author” section, enter your conflict of interest statement in the “Confidential to Editor” section, and submit your "Accept" recommendation.

Reviewer #1: (No Response)

2. Is the manuscript technically sound, and do the data support the conclusions?

The manuscript must describe a technically sound piece of scientific research with data that supports the conclusions. Experiments must have been conducted rigorously, with appropriate controls, replication, and sample sizes. The conclusions must be drawn appropriately based on the data presented.

Reviewer #1: Yes

3. Has the statistical analysis been performed appropriately and rigorously?

Reviewer #1: N/A

4. Have the authors made all data underlying the findings in their manuscript fully available?

The [PLOS Data policy](http://www.plosone.org/static/policies.action#sharing) requires authors to make all data underlying the findings described in their manuscript fully available without restriction, with rare exception (please refer to the Data Availability Statement in the manuscript PDF file). The data should be provided as part of the manuscript or its supporting information, or deposited to a public repository. For example, in addition to summary statistics, the data points behind means, medians and variance measures should be available. If there are restrictions on publicly sharing data—e.g. participant privacy or use of data from a third party—those must be specified.

Reviewer #1: Yes

5. Is the manuscript presented in an intelligible fashion and written in standard English?

PLOS ONE does not copyedit accepted manuscripts, so the language in submitted articles must be clear, correct, and unambiguous. Any typographical or grammatical errors should be corrected at revision, so please note any specific errors here.

Reviewer #1: Yes

6. Review Comments to the Author

Please use the space provided to explain your answers to the questions above. You may also include additional comments for the author, including concerns about dual publication, research ethics, or publication ethics. (Please upload your review as an attachment if it exceeds 20,000 characters)

**Reviewer #1: General Comments:**
The manuscript is an interesting analysis of the genes identified as responsible for metabolism-based NTS herbicide resistance in plants. The manuscript is well-written and should be of interest to a broad range of plant scientists.

Specific Comments/Questions:
The amendments to the tables, especially table 1, were useful in clarifying the relationship between the species selected for analysis. Major concerns of the reviewers appear to be addressed.

One area that is still unclear is where the diversity occurs among these genes identified as being responsible for herbicide metabolism (e.g., promoter region, substrate binding site). Supplementary figures 3 and 4 show sequence alignment, but could a summary statement as to how CYPs within a clan and GSTs within a class differ (i.e., what are the criteria used to distinguish one from another)? Are most of the differences in the substrate binding regions or in regulatory regions or both?

Response:

Classification of CYPs into clans is based on their position on the tree. The variation in CYP protein sequences within a clan can be very high (less than 40% identity) throughout the alignment, except in the heme-binding, oxygen binding, and ERR triad domains which are more conserved. More variable regions include the membrane targeting region and substrate recognition sites. Sequence variation between clan members is higher in the larger clans.

GSTs are classified into classes based on their sequence identity and kinetic properties. There is very large sequence diversity within classes in plants (can have lower than 40% amino acid identity within a class). The N-terminal domain containing the GSH-binding sites is usually more conserved and the C-terminal domain which contains most of the substrate recognition sites is more variable.

Additional information on GST class and CYP clan/family classification has been provided in lines 69, and 91-94. An overview of protein features of GSTs has been added to S1 Figure.

Minor Suggestions/Corrections:
Introduction
Lines 63 & 64: Capitalize and italicize the “s” in glutathione *S*-transferases.
Line 75: Space needed between “mitochondrial,or”.
Lines 102-107: The added sentences need a reference.

Response: The corrections have been made to the text.

Materials & Methods
Lines 145-146: Why was the P. patens sequence used with A. thaliana and O. sativa for the GST BLASTP searches and not in the CYP BLASTP searches?

Response: *A. thaliana* and *O. sativa* CYP sequences were sufficient for the BLASTP searches to retrieve all CYP sequences in other species.

In 2013 new plant GST classes were discovered in the genome of *P. patens* that do not exist in *A. thaliana* and *O. sativa* (hemerythrin, Iota and Ure2p) (Liu et al., 2013). The Hemerythrin and Iota GSTs have large class-specific protein domains and low amino acid sequence identity with sequences in other GST classes. For example, there is just 10% amino acid sequence identity between Pp3c3_21020V3.1 (PpGSTH1) and Pp3c15_23900V3.1 (PpGSTF1). To ensure that members of these classes were identified in the other species, *P. patens* GSTs were used as queries in addition to *A. thaliana* and *O. sativa* GSTs for BLASTP searches against the genomes of the other 8 species.

Liu, Y. et al. Functional divergence of the glutathione S-transferase supergene family in *Physcomitrella patens* reveals complex patterns of large gene family evolution in land plants. (2013). Plant Physiology. 161(2): 773-86. doi: 10.1104/PP.112.205815.

Line 156: Include “(2N)” after the words “N-terminal domains” to identify 2N.

Response: the correction has been made to the text.

Lines 153-158: The identifiers for the mitochondrial and microsomal GST classes do not match those in Lines 81-83 (Kappa vs metaxin class and MAPEG vs mPGES2 class).

Response: These are four different groups of proteins. Kappa and MAPEG proteins are sometimes called GSTs because they exhibit glutathione transferase activity, however they do not possess the characteristic GST domain architecture, and in the case of kappa GSTs, are more closely related to a bacterial protein disulphide bond isomerase (dsbA) than to other GST classes (Ladner et al., 2004).

I have rewritten the sentence to include this information and referenced the two below papers that characterised kappa and MAPEG enzymes:

Ladner, J., Parsons, J., et al. (2004). Parallel evolutionary pathways for glutathione transferases: structure and mechanism of the mitochondrial class kappa enzyme rGSTK1-1. Biochemistry. 43:352-61. doi: 10.1021/bi035832z.

Bresell, A., Weinander, R. et al. (2005). Bioinformatic and enzymatic characterization of the MAPEG superfamily. The FEBS Journal. 272:1688-1703. doi: 10.1111/J.1742-4658.2005.04596.X

Results
Lines 204-205: Why the distinction for the *M. polymorpha* CYP sequences here and no mention of other species?

Response: This is because they were named for this paper by Dr David Nelson who developed the naming system for cytochrome P450s.

Line 254: Maybe change “and” to “or” as all 6 species are not covered in each of the 4 (711, 727, 746, 747) of the 12 clans.

Response: This has been corrected in the text.

Lines 285 & 286: Should clan 741 be included in these two lists?

Response: Yes it should, it has been included.

Line 324: For consistency, make streptophytes lower case.
Line 461: Reference Table 2 instead of S3.
Line 521: For completeness, add HRAC group for triazines.

Response: the suggested changes have been made to the text.

Line 522: From which table or reference is the 61-80% derived?

Response: 61-80% is from the total number of GSTs identified in this study belonging to the tau and phi classes in *S. moellendorffii*, *O. sativa* and *A. thaliana* (61% in Sm, 67% in At, 80% in Os). The sentence has been rewritten for clarity.

Discussion
Line 566: Is “mutation” the correct word here? Isn’t it possible that these enzymes naturally metabolize the herbicide and that duplication of genes or copy numbers upon selection pressure confers the resistance?

Response: this is true, the sentence has been reworded.

Figures/Tables
Figure 1: It would be useful to add in the legend that chlorophyte is synonymous with streptophyte. Figures 1 & 2 use chlorophyte while the description in the results uses the streptophyte term.

Response: charophyte is synonymous with streptophyte algae. This has been included in the Fig 1 legend.

Figure 2C: Should “phi” be included in the most wide-ranging list (all Archaeplastida) or with lambda, tau, and ure2p (land plants and charophytes/streptophytes)? The text in lines 359-361 indicates that “phi” should not be in the all-Archaeplastida list.

Response: this is correct and was written in error. Phi has been removed from the ‘all Archaeplastida’ list.

Table 3: Spelling of sulfonylurea varies between Tables 2 & 3.

Response: thank you for noticing this, the spelling has been changed to be sulfonylurea.

7. PLOS authors have the option to publish the peer review history of their article ([what does this mean?](https://journals.plos.org/plosone/s/editorial-and-peer-review-process#loc-peer-review-history)). If published, this will include your full peer review and any attached files.


If you choose “no”, your identity will remain anonymous but your review may still be made public.
